# Combined Drought and Heat Activates Protective Responses in *Eucalyptus globulus* That Are Not Activated When Subjected to Drought or Heat Stress Alone

**DOI:** 10.3389/fpls.2018.00819

**Published:** 2018-06-20

**Authors:** Barbara Correia, Robert D. Hancock, Joana Amaral, Aurelio Gomez-Cadenas, Luis Valledor, Glória Pinto

**Affiliations:** ^1^Department of Biology, Centre for Environmental and Marine Studies, University of Aveiro, Aveiro, Portugal; ^2^Cell and Molecular Sciences, The James Hutton Institute, Dundee, United Kingdom; ^3^Departamento de Ciencias Agrarias y del Medio Natural, Universitat Jaume I, Castellón de la Plana, Spain; ^4^Department of Organisms and Systems Biology, University of Oviedo, Oviedo, Spain

**Keywords:** plant metabolism, isolated stress, combined stress, recovery, network analysis

## Abstract

Aiming to mimic a more realistic field condition and to determine convergent and divergent responses of individual stresses in relation to their combination, we explored physiological, biochemical, and metabolomic alterations after drought and heat stress imposition (alone and combined) and recovery, using a drought-tolerant *Eucalyptus globulus* clone. When plants were exposed to drought alone, the main responses included reduced pre-dawn water potential (Ψ_pd_) and gas exchange. This was accompanied by increases in malondialdehyde (MDA) and total glutathione, indicative of oxidative stress. Abscisic acid (ABA) levels increased while the content of jasmonic acid (JA) fell. Metabolic alterations included reductions in the levels of sugar phosphates accompanied by increases in starch and non-structural carbohydrates. Levels of α-glycerophosphate and shikimate were also reduced while free amino acids increased. On the other hand, heat alone triggered an increase in relative water content (RWC) and Ψ_pd_. Photosynthetic rate and pigments were reduced accompanied by a reduction in water use efficiency. Heat-induced a reduction of salicylic acid (SA) and JA content. Sugar alcohols and several amino acids were enhanced by the heat treatment while starch, fructose-6-phosphate, glucose-6-phosphate, and α-glycerophosphate were reduced. Contrary to what was observed under drought, heat stress activated the shikimic acid pathway. Drought-stressed plants subject to a heat shock exhibited a sharp decrease in gas exchange, Ψ_pd_ and JA, no alterations in electrolyte leakage, MDA, starch, and pigments and increased glutathione pool in relation to control. Comparing this with drought stress alone, subjecting drought stressed plants to an additional heat stress alleviated Ψ_pd_ and MDA, maintained an increased glutathione pool and reduced starch content and non-structural carbohydrates. A novel response triggered by the combined stress was the accumulation of cinnamate. Regarding recovery, most of the parameters affected by each stress condition reversed after re-establishment of control growing conditions. These results highlight that the combination of drought and heat provides significant protection from more detrimental effects of drought-stressed eucalypts, confirming that combined stress alter plant metabolism in a novel manner that cannot be extrapolated by the sum of the different stresses applied individually.

## Introduction

Forest trees, as all sessile plants, have evolved many mechanisms that enable them to thrive in variable environmental conditions, ranging from circadian regulation (Dodd et al., 2005) to recovery from overpowering stress (Brodribb and Cochard, 2009). Despite these physiological adaptations, the long life-span of trees does not allow for rapid genetic adaptation to environmental changes, rendering forests particularly susceptible to climate change (Lindner et al., 2010). Therefore, climate-driven forest vulnerability and tree die-off have become emerging concerns for forest sustainability worldwide (Anderegg et al., 2012; Allen et al., 2015).

Decades of research have significantly improved our understanding of how abiotic stresses that plants encounter in the field, such as drought and heat stress, affect plant development and growth (Rennenberg et al., 2006). However, predominant abiotic stress factors have been mostly tested individually and under controlled laboratory conditions (Mittler and Blumwald, 2010). In contrast, relatively little attention has been given to the combined effects of abiotic stresses, for example, in the field water deficit does not occur alone but associated with high temperature or high light (Chaves et al., 2002).

There is a growing body of evidence that the impacts of a combination of different stress factors on plant functioning traits do not necessarily lead to an additive response but rather to unique responses as a consequence of a synergistic or antagonistic effect of both stress factors (Bansal et al., 2013; Pandey et al., 2015). The high degree of complexity results from the fact that when two stresses co-occur, plant adaptation to the stress combination is governed by the interaction of the two stresses, controlled by different signaling pathways that may interact, inhibit one another or be prioritized differentially by the plant (Zandalinas et al., 2017).

A particular abiotic stress induces a plant response tailored to that specific environmental condition and, when encountering different combined stresses, a plant might actually require conflicting adjustments (Mittler and Blumwald, 2010). Under combined drought and heat stress, for example, plants have to act and balance stomatal responses between preventing water loss and cooling their leaves by transpiration, meaning that a proper defense response depends simultaneously on decreasing and increasing stomatal conductance (Mittler and Blumwald, 2010).

The previous example leaves no doubt on the challenging task of researching abiotic stress combination. Several studies have already researched this subject mainly focusing on the drought and heat combination (Valladares and Pearcy, 1997; Dobra et al., 2010; Silva et al., 2010; Arend et al., 2013; Duan et al., 2014). The results indicate a plethora of plant responses ranging from stomatal and non-stomatal limitations to photosynthesis (Arend et al., 2013), photo-inhibition (Valladares and Pearcy, 1997), changes in key stress signaling components, such as reactive oxygen species (Silva et al., 2010) and plant hormones (Dobra et al., 2010), up to rapid mortality through loss of stem hydraulic conductivity (Duan et al., 2014). Furthermore, the conclusions are very species/experiment dependent: elevated temperature is beneficial when imposed alone but is detrimental when combined with drought (Arend et al., 2013); elevated temperature triggers rapid mortality through hydraulic failure, which is induced by drought (Duan et al., 2014); drought greatly disturbs photosystem II activity and oxidative metabolism, which are strongly stimulated by heat stress (Silva et al., 2010). Given the known impact of abiotic stress on the plant metabolome (Warren et al., 2011; Hochberg et al., 2013), we would also expect extensive research on this topic, but the available knowledge is limited (Obata et al., 2015).

Among forest plantations, *Eucalyptus* species play an increasingly essential role to guarantee the world’s demand for wood products, and assessing the impact of drought and heat on such economically important plants is highly pertinent since both factors are considered the main drivers controlling vulnerability of *Eucalyptus* plantations (Booth, 2013). Our previous research which compared results from a controlled climate chamber experiment with field-grown *Eucalyptus globulus* Labill. corroborated that the knowledge acquired from imposing the stress individually to test stress-tolerant plants cannot be extrapolated to field-grown plants (Correia et al., 2018). This urged us to evaluate the impact of combined drought and heat stress in *E. globulus* plants, mimicking a more realistic field condition. Since assessing recovery may also be very informative and provide better insights of the severity of the combined stress than observations done at the stress imposition (Mitchell et al., 2013), we have also included a post stress period.

This study hence arises from the need to elucidate the major responses that take place in *E. globulus* under combined drought and heat stress. Aiming to determine convergent and divergent responses of the individual stress in relation to their combination, we explored physiological and biochemical alterations after stress imposition (alone and combined) and recovery using a drought-tolerant clone. An additional key goal was to get an extra dimension by identifying and integrating major metabolomic alterations.

## Materials and Methods

### Plant Material and Experimental Design

Rooted cuttings of *E. globulus* (clone AL-18) were obtained from the breeding program of Altri Florestal SA (Portugal) and transplanted to 1 L plastic pots filled with equal weight of a 3:2 (w/w) peat:perlite mixture. The potted cuttings were then divided and placed in two climate chambers (Fitoclima 1200, Aralab, Portugal) for a one-month acclimation period. Conditions were 25/20°C (day/night), 16/8 h (day/night) photoperiod, 50% relative humidity and 600 μmol m^-2^ s^-1^ photosynthetic photon flux density. During the acclimation period, plants were watered up to 70% field capacity (FC) and fertilized weekly with a NPK (5:8:10) nutritive solution. Pot weight was monitored every day and the percentages of FC were maintained by adding the amount of water lost. During the experiment, environmental conditions inside the climate chambers were maintained as in the acclimation period and only watering was altered. Half of the cuttings in each climate chamber was assigned to a control well-watered regime (C: water supplied every day until soil water content reached 70% FC) and the other half was assigned to a drought regime (D: water supplied every day until soil content reached 18% FC). This lasted for 5 days. Air temperature inside the second climate chamber was then gradually increased, and plants from both groups (C and D) were subject to 40°C during 4 h (H – heat stress; and D^∗^H – combined (combination of both drought and heat) stress, respectively). At this moment, the first sampling took place: C and D plants were sampled from the first climate chamber, H, and D^∗^H plants were sampled from the second climate chamber. In order to perform a realistic experiment, corresponding with the dawn, heat exposure treatment began with an increasing temperature gradient from 20 to 40°C for 3 h, which was then maintained for 4 h. Lightweight expanded clay aggregate (LECA^®^), together with a refrigeration system, was used around the pots in order to mimic a fresher field soil temperature under heat stress. After that, environmental conditions inside the climate chambers were restored and all cuttings were well-watered (70% FC). The recovery of all groups was then evaluated at the second sampling point after 4 days under environmental and watering control conditions. In order to minimize the effects of environmental heterogeneity, the pots were periodically moved to the neighboring position during the whole experiment.

At each sampling point (first sampling point: stress; second sampling point: recovery), five plants per group (i.e., C, D, H, and D^∗^H) were used to evaluate plant water potential. Homogenous leaves from six individuals were used for *in vivo* measurements of leaf gas exchange parameters and subsequently used to determine plant relative water content (RWC) and electrolyte leakage. Also, homogeneous leaves from six individuals were immediately frozen in liquid nitrogen and kept at -80°C for further analysis (lipid peroxidation, redox couples ascorbate and glutathione, quantification of starch and pigments, hormonal alterations and metabolomics).

### Water Relations

Predawn water potential (Ψ_pd_) was measured using a Scholander-type pressure chamber (PMS Instrument Co., Corvallis, OR, United States). Four leaf discs (diameter = 11 mm) per individual were also collected to determine RWC, by using the equation: RWC = (FW-DW)/(TW-DW) × 100, in which FW is the fresh weight, TW is the turgid weight after rehydration of the leaf discs for 24 h at 4°C in the dark, and DW is the dry weight after oven-drying the leaf discs at 70°C until they reached a constant weight.

### Gas Exchange and Stomatal Conductance

Leaf gas exchange measurements were performed on fully expanded leaves using an infrared gas analyzer, LCpro-SD (ADC BioScientific Ltd., United Kingdom), equipped with the broad leaf chamber. Measurements were performed maintaining the following conditions inside the chamber: ambient temperature, CO_2_ and H_2_O concentration, air flow 200 μmol s^-1^ and light intensity 400 μmol m^-2^ s^-1^. Data were recorded when the measured parameters were stable (2–6 min). Net CO_2_ assimilation rate (A), transpiration rate (E), stomatal conductance (g_s_), and internal CO_2_ concentration (C_i_) were determined. Water use efficiency (WUE) was calculated based on leaf gas exchange, using the formulae WUE = A/E.

### Starch Quantification

Starch concentration was determined by using the anthrone method. Total soluble sugars were extracted from 50 mg of frozen leaves in 80% (v/v) ethanol for 1 h at 80°C. After centrifugation, the pellet was used to quantify starch, as described by Osaki et al. (1991). The pellet was resuspended with 30% (v/v) perchloric acid and incubated at 60°C for 1 h. The mixture was then centrifuged and anthrone was added to the supernatant. After heating the mixture at 100°C for 10 min, absorbance was read at 625 nm (Thermo Fisher Scientific Spectrophotometer, Genesys 10-uv S) and starch concentration was determined according to a D-glucose standard curve.

### Pigments Quantification

Concentration of chlorophyll a, b, and carotenoids was determined according to Sims and Gamon (2002). Pigments were extracted using cold acetone:50 mM Tris buffer pH 7.8 (80:20) (v/v). Following centrifugation, supernatant absorbance was read at 470, 537, 647, and 663 nm (Thermo Fisher Scientific Spectrophotometer, Genesys 10-uv S). Chlorophyll a, b, and carotenoids were then quantified by using the formulae presented by the author.

### Electrolyte Leakage and Lipid Peroxidation

To determine electrolyte leakage (EL), four leaf discs (diameter = 11 mm) were collected. Conductivity was measured (CONSORT C830, Consort bvba, Turnhout, Belgium) and EL was determined using the equation: EL = (C_i_ - W_c_)/(C_f_ - W_c_) × 100, in which W_c_ represents water conductivity, C_i_ is the initial conductivity of water plus the leaf discs, and C_f_ is the final conductivity of water plus the leaf discs after 5 min at 121°C and 24 h at 4°C.

The extent of lipid peroxidation on leaves was estimated by measuring the amount of malondialdehyde (MDA), following an adaptation of the procedure described by Hodges et al. (1999). About 100 mg of leaves were ground in 2.5 mL of cold 0.1% (w/v) trichloroacetic acid (TCA) and centrifuged. A 250 μL aliquot of the supernatant was added to 1 mL of 20% (w/v) TCA containing 0.5% (w/v) TBA (positive control), and another 250 μL was added to 1 mL of 20% (w/v) TCA (negative control). Both positive and negative controls per sample were heated at 95°C for 30 min. After stopping the reaction on ice, absorbance was read at 440, 532, and 600 nm (Thermo Fisher Scientific Spectrophotometer, Genesys 10-uv S, Waltham, MA, United States), and MDA content was determined by the formulae presented by the author.

### Non-protein Redox Couples Ascorbate and Glutathione

Ascorbate (reduced, AsA) and dehydroascorbate (DHA) concentrations, as well as oxidized (GSSG) and reduced (GSH) glutathione were determined according to the microplate method described by Queval and Noctor (2007).

### Hormone Quantification

Abscisic acid (ABA), jasmonic acid (JA), and salicylic acid (SA) were extracted and analyzed following the procedure described by Durgbanshi et al. (2005), with slight modifications. Freeze-dried tissue (50 mg) was mixed with 100 ng of ABAd_6_, 100 ng of SAd_6_ and 100 ng of dihydrojasmonic acid and homogenized with 5 mL of distilled water. After cold centrifugation, supernatants were recovered and pH adjusted to 3 with 30% acetic acid. The acidified water extract was partitioned twice against 3 mL of diethyl ether. The organic upper layer was recovered and vacuum evaporated in a centrifuge concentrator (SpeedVac, Jouan, Saint Herblain, France). The dry residue was then resuspended in a 10% methanol solution by gentle sonication. The resulting solution was passed through 0.22 μm regenerated cellulose membrane syringe filters (Albet S.A., Barcelona, Spain) and directly injected into a UPLC system (Acquity SDS, Waters Corp., Milford, MA, United States). Analytes were separated by reversed-phase (Nucleodur C18, 1.8 μm 50 × 2.0 mm, Macherey-Nagel, Barcelona, Spain) using a linear gradient of ultrapure water (A) and methanol (B) (both supplemented with 0.01% acetic acid) at a flow rate of 300 μL min^-1^. The gradient used was: (0–2 min) 90:10 (A:B), (2–6 min) 10:90 (A:B) and (6–7 min) 90:10 (A:B). Hormones were quantified with a Quattro LC triple quadrupole mass spectrometer (Micromass, Manchester, United Kingdom) connected online to the output of the column through an orthogonal Z-spray electrospray ion source. The analytes were quantified after external calibration against the standards.

### Metabolomics Analysis

Metabolites were extracted, derivatized and analyzed by gas chromatography-mass spectrometry (GC-MS), as previously described by Foito et al. (2013). *Eucalyptus* leaves were lyophilized and 100 mg of dried, powdered material were weighed into glass tubes. Lyophilized material was extracted sequentially in methanol, water and chloroform for 30 min at 30°C each. Internal standards (aqueous ribitol and methanolic *n*-non-adecanoic acid) were added during the initial methanol extraction step. Finally, an additional aliquot of water was added and the polar and non-polar phases were separated, evaporated to dryness and derivatized independently. Metabolite profiles of the polar and non-polar fractions were acquired following separation of compounds on a DB5-MSTM column (15 m × 0.25 mm × 0.25 μm; J&W, Folsom, CA, United States) using a Thermo-Finnigan DSQ II GC-MS system (Thermo Finnigan, United Kingdom). The samples were analyzed as a single batch, in a randomized order, while quality control samples as well as blanks were incorporated at the beginning and the end of the sequence. Peak areas were calculated in relation to respective internal standard and normalized to respective extracted weight. Metabolites were identified based on their mass spectral characteristics and GC retention times, by comparison with retention times of reference compounds from an in-house reference library as previously described (Correia et al., 2016b).

### Statistical Analysis

Data are presented as mean ± SE (standard error) of three to six independent biological replicates. Statistical procedures were performed using SigmaPlot for Windows v. 11.0 (Systat Software Inc., San Jose, CA, United States), except metabolites that were analyzed using GenStat v16 (VSN International Ltd., Hemel Hempstead, United Kingdom). One-way analysis of variance (ANOVA) followed by the Fisher’s LSD *post hoc* all pairwise multiple comparison tests were employed separately for each sampling point (i.e., stress and recovery) to estimate the significance of the results. Different lower cases indicate significant differences between treatments (C, D, H, and D^∗^H) at *p* ≤ 0.05. In order to integrate the results, a complete dataset comprising all physiological, biochemical and metabolomic data was subjected to principal component analysis (PCA), sparse partial least squares (sPLS) and network analyses using the software R v3.1.2 core functions (R Core Team, 2014) plus the package mixOmics (Lê Cao et al., 2016). For building the sPLS model, the performance was first evaluated over 10 components, and two components (total Q2 > 0.1) were selected. Variables were then selected according to individual Q2, and those variables with a value lower than 0.1 were filtered out to prevent later overfitting of the model. The network was plotted employing the mixOmics network function, establishing a cut-off of 0.65 (which roughly correspond to plotting the variables with a Q2 > 0.35 for at least one of the two components).

## Results

**FIGURE 1:**
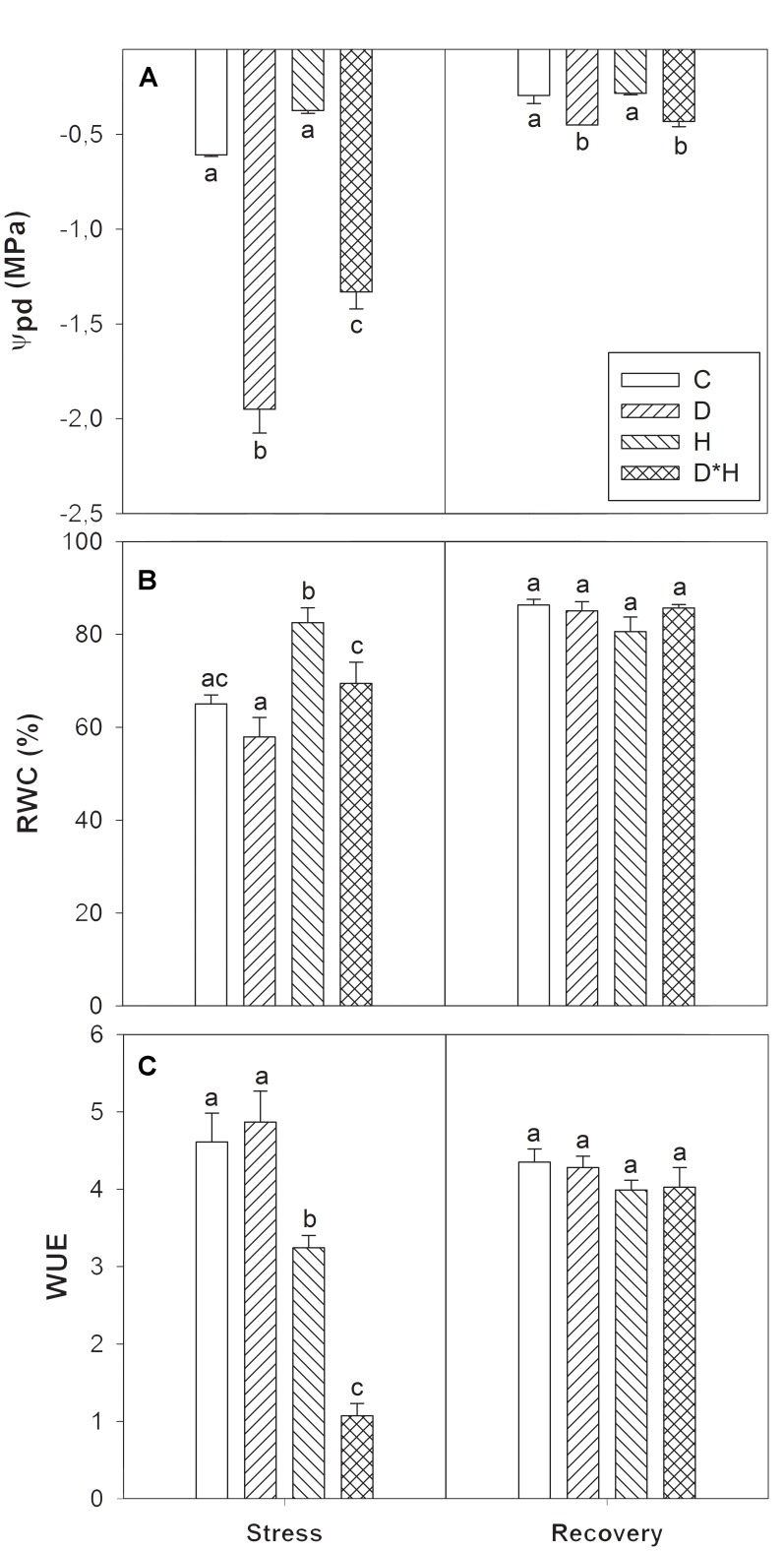
Predawn shoot water potential (Ψ_pd_, **A**); relative water content (RWC, **B**), and water use efficiency (WUE, **C**) of *Eucalyptus globulus* exposed to control conditions (C), drought (D), heat (H), and combined stress (D^∗^H) and respective recovery. Different lowercase letters indicate significant differences among the four treated groups (C, D, H and D^∗^H), after stress and after recovery, separately (*p* ≤ 0.05).

The effect of drought (D) and heat (H) stress applied alone and combined (D^∗^H) in *E. globulus* plants was analyzed by assessing physiological, biochemical, hormonal and metabolomic alterations after stress imposition and recovery. The plant water status was evaluated by Ψ_pd_, RWC and WUE. Drought and combined stress induced a significant reduction in Ψ_pd_, with the extent of reduction being higher in the drought stress alone than in the combined stress (**Figure 1A**). After recovery, although increased, Ψ_pd_ of drought and combined stress was still lower than the control (**Figure 1A**). RWC was only slightly decreased after the drought treatment (not significant), significantly increased in heat, and was unaffected in the combined stress (**Figure 1B**). Finally, WUE revealed a reduction in heat and combined stress, with the combined stress exhibiting the most severe reduction in WUE (**Figure 1C**). Both RWC and WUE fully recovered after returning to control conditions (**Figures 1B,C**).

**FIGURE 2:**
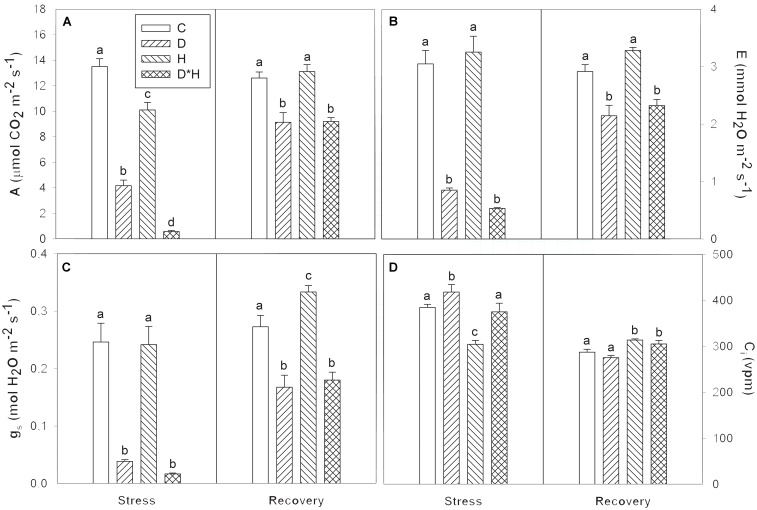
Net photosynthetic rate (A, **A**), transpiration rate (E, **B**), stomatal conductance (g_s_, **C**), and internal CO_2_ concentration (C_i_, **D**) of *Eucalyptus globulus* exposed to control conditions (C), drought (D), heat (H), and combined stress (D^∗^H) and respective recovery. Different lowercase letters indicate significant differences among the four treated groups (C, D, H, and D^∗^H), after stress and after recovery, separately (*p* ≤ 0.05).

Gas exchange varied in response to the imposed stresses (**Figure 2**). Net photosynthetic rate (A) was reduced following all stress treatments (**Figure 2A**) with the combined stress leading to the greatest reduction, followed by drought and heat stress. Transpiration rate (E) and stomatal conductance (g_s_) were similarly affected with only drought and combined stress resulting in a decrease (**Figures 2B,C**). On the other hand, internal CO_2_ concentration (C_i_) significantly increased in drought, and decreased in heat, remaining unchanged in the combined stress (**Figure 2D**). Most of these responses only slightly leveled off following recovery (**Figures 2A–C**).

**FIGURE 3:**
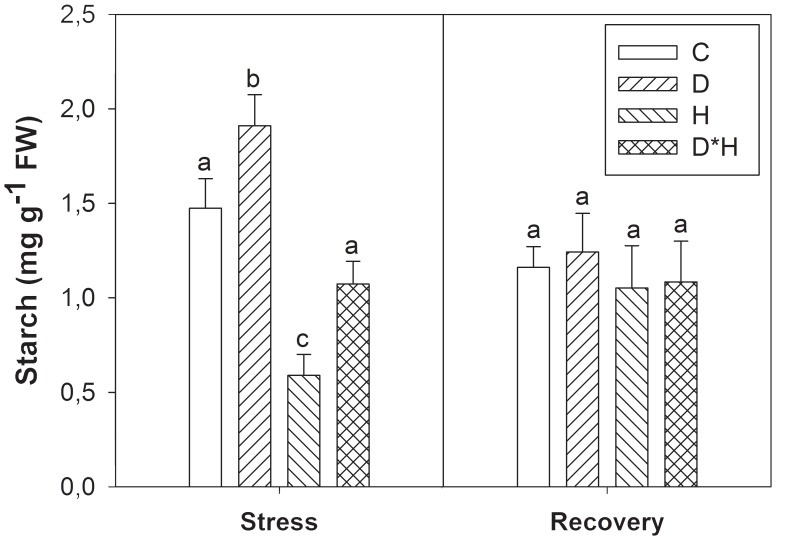
Starch of *Eucalyptus globulus* exposed to control conditions (C), drought (D), heat (H), and combined stress (D^∗^H) and respective recovery. Different lowercase letters indicate significant differences among the four treated groups (C, D, H and D^∗^H), after stress and after recovery, separately (*p* ≤ 0.05).

Leaf starch content increased in drought, decreased in heat and slightly although not significantly decreased in the combined stress. After recovery leaf starch was similar regardless of prior treatment (**Figure 3**).

**FIGURE 4:**
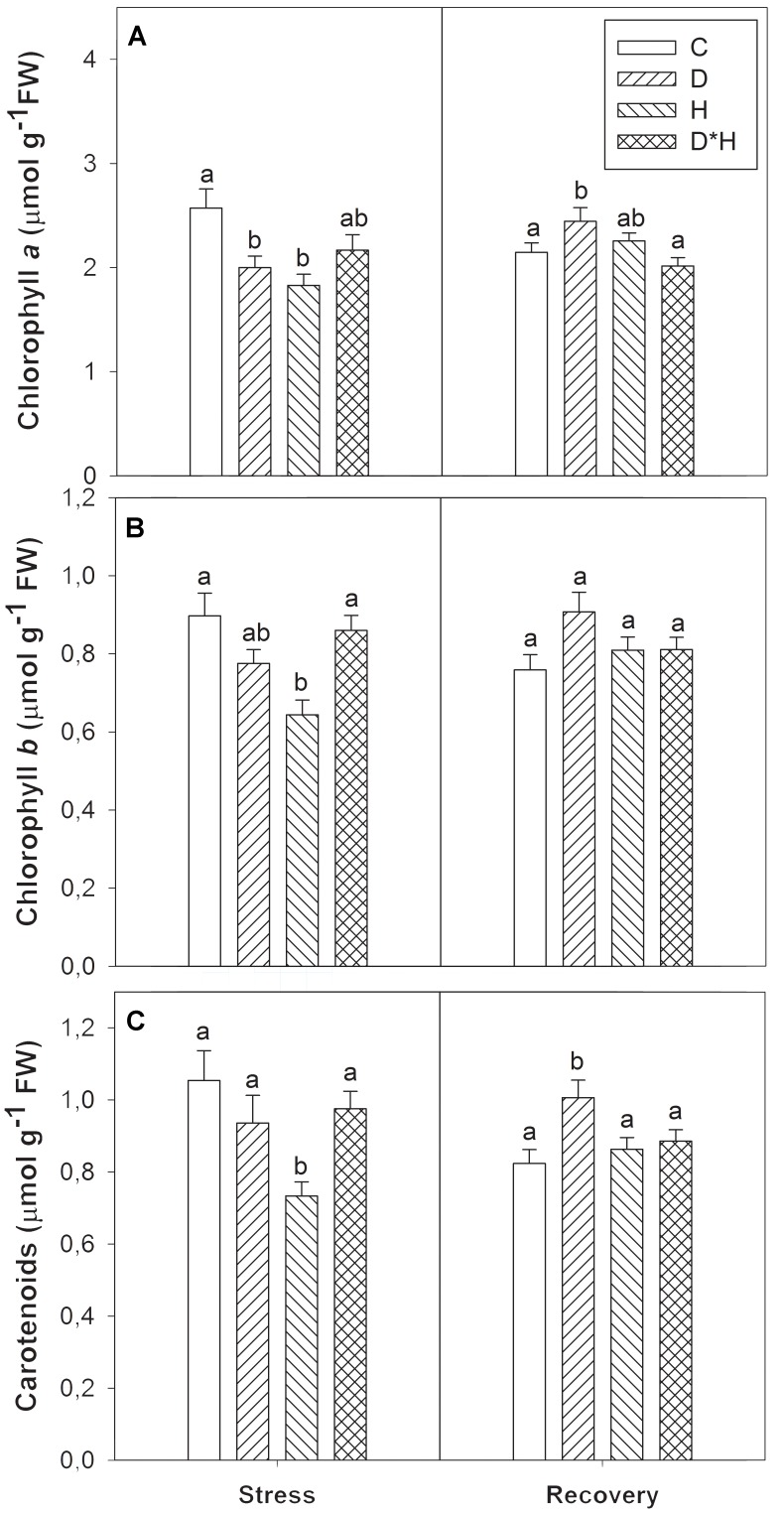
Chlorophyll a **(A)**, chlorophyll b **(B)**, and carotenoids **(C)** of *Eucalyptus globulus* exposed to control conditions (C), drought (D), heat (H), and combined stress (D^∗^H) and respective recovery. Different lowercase letters indicate significant differences among the four treated groups (C, D, H, and D^∗^H), after stress and after recovery, separately (*p* ≤ 0.05).

Chlorophyll a, b, and carotenoids were differentially modulated by the imposed stresses (**Figure 4**). Chlorophyll a decreased in drought and heat (**Figure 4A**) while chlorophyll b was only reduced after the heat treatment (**Figure 4B**). After recovery, chlorophyll a was higher in previously drought stressed plants than in control plants (**Figure 4A**). The carotenoid abundance profile matched chlorophyll b exhibiting a major reduction only in the heat stress and an increase in drought stressed plants after recovery (**Figure 4C**).

**FIGURE 5:**
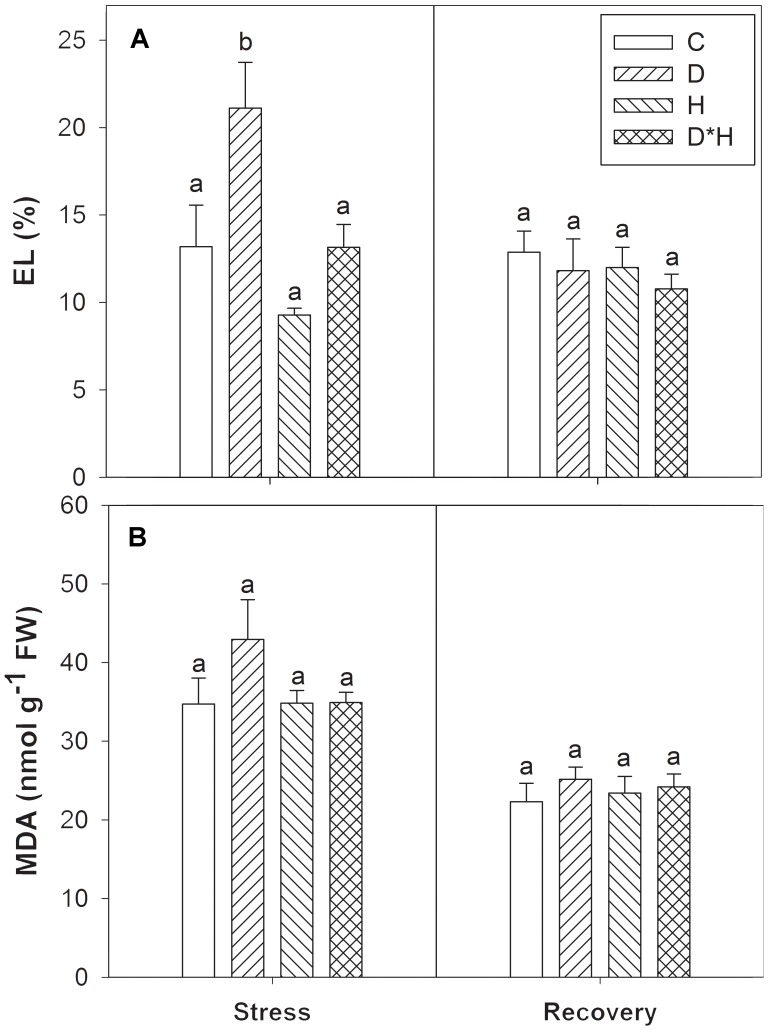
Electrolyte leakage (EL, **A**) and malondialdehyde content (MDA, **B**) of *Eucalyptus globulus* exposed to control conditions (C), drought (D), heat (H), and combined stress (D^∗^H) and respective recovery. Different lowercase letters indicate significant differences among the four treated groups (C, D, H, and D^∗^H), after stress and after recovery, separately (*p* ≤ 0.05).

Membrane integrity was assessed by leaf EL and MDA accumulation. Electrolyte leakage revealed a significant increase during drought stress (**Figure 5A**) that was accompanied by a trend towards higher MDA at the same point (**Figure 5B**). After recovery, all plants exhibited equivalent EL and MDA content (**Figures 5A,B**).

**FIGURE 6:**
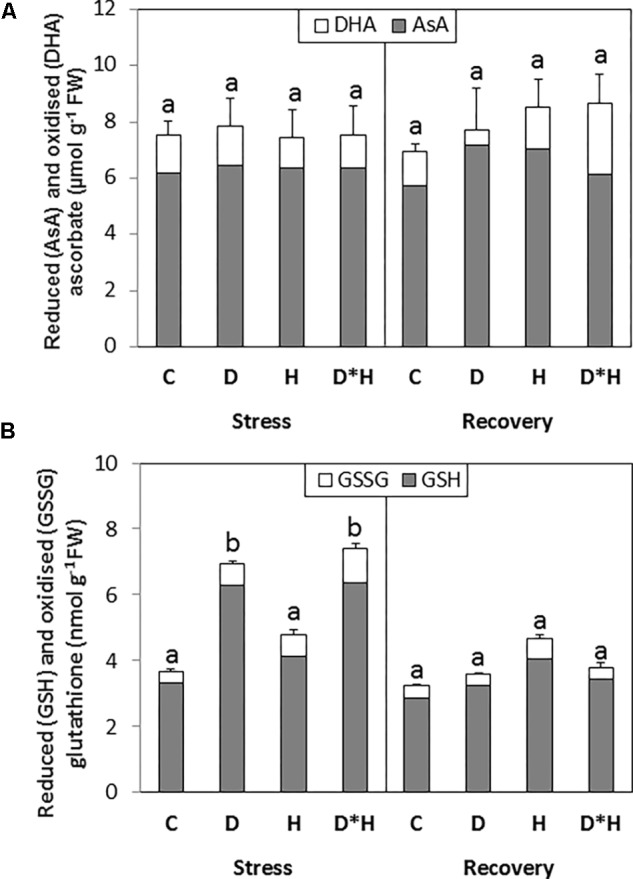
Total ascorbate [incorporating reduced (AsA) and oxidized (DHA) forms; **A**] and total glutathione [incorporating reduced (GSH) and oxidized (GSSG) forms; **B**] quantified in the leaves of *Eucalyptus globulus* exposed to control conditions (C), drought (D), heat (H), and combined stress (D^∗^H) and respective recovery. Different lowercase letters indicate significant differences in the total AsA or GSH pools among the four treated groups (C, D, H, and D^∗^H), after stress and after recovery, separately (*p* ≤ 0.05).

The total AsA pool was not affected by the imposed stresses; on the contrary the total glutathione pool was increased in the drought and combined stress (**Figure 6**). This induction was not accompanied by an increase in the oxidized pool (**Figure 6**). After recovery, an increase in the oxidation status of AsA pool was observed without major alterations in the total AsA content. Glutathione content of leaves subjected to drought or combined stress return to control levels following recovery (**Figure 6**).

**FIGURE 7:**
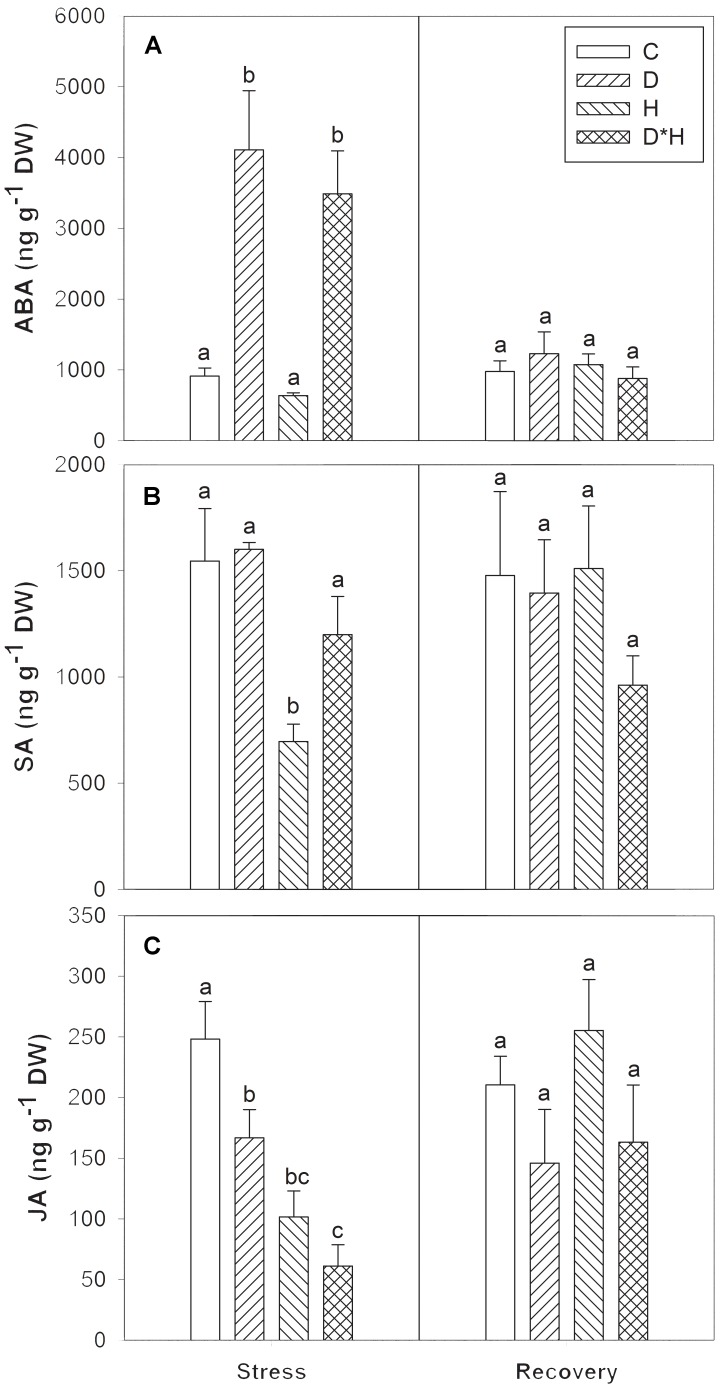
Abscisic acid (ABA, **A**), salicylic acid (SA, **B**), and jasmonic acid (JA, **C**) of *Eucalyptus globulus* exposed to control conditions (C), drought (D), heat (H), and combined stress (D^∗^H) and respective recovery. Different lowercase letters indicate significant differences among the four treated groups (C, D, H, and D^∗^H), after stress and after recovery, separately (*p* ≤ 0.05).

The imposed stresses significantly affected the leaf hormonal dynamics and major differences were found regarding ABA, SA, and JA, as shown in **Figure 7**. On one hand, ABA significantly accumulated in drought and combined stress (**Figure 7A**). On the other hand, SA levels decreased exclusively after heat (**Figure 7B**) and JA content decreased under all stress conditions in a descending order: drought, heat, and combined stress (**Figure 7C**). No hormonal differences were detected after recovery from stress.

The foliar metabolite profile of *E. globulus* subjected to drought, heat, combined stress and recovery were compiled using GC–MS. This analysis yielded the detection of 106 metabolites (Supplementary Table S1), distinguishing between 64 polar and 42 non-polar metabolites. Only a small part of the detected metabolites could not be identified after data processing (7 polar and 5 non-polar metabolites). From the identified metabolites, 48 showed significant changes due to the applied stress and/or recovery, including 12 carbohydrates (**Table 1**), 5 organic acids and 17 amino acids (**Table 2**), 2 phenolic acids, 6 fatty acids/alcohols, 1 phytosterol, and 5 unknown metabolites (**Table 3**).

**Table 1:** Metabolomic analysis, relative abundance of carbohydrates.

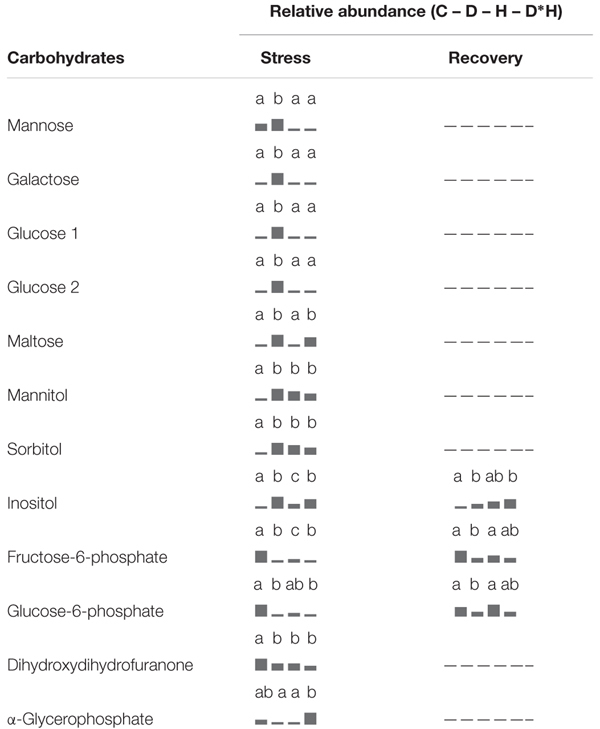

*Abundance data is presented on a scale relative to the lowest value among treatments. Different lowercase letters indicate significant differences between the control (C), drought (D), heat (H), and combined stress (D^∗^H) plant groups, under stress and after recovery; absent relative abundance indicates that no differences were found (*p* ≤ 0.05).*

**Table 2:** Metabolomic analysis, relative abundance of organic acids and amino acids.

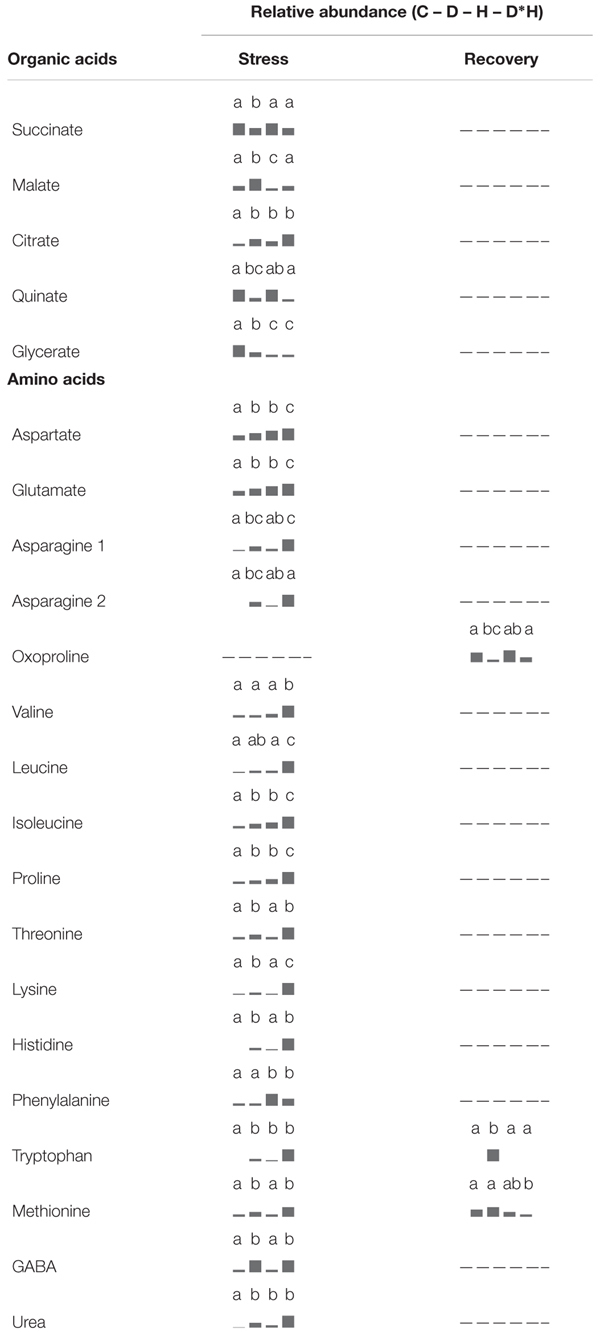

*Abundance data is presented on a scale relative to the lowest value among treatments. Different lowercase letters indicate significant differences between the control (C), drought (D), heat (H), and combined stress (D^∗^H) plant groups, under stress and after recovery; absent relative abundance indicates that no differences were found (*p* ≤ 0.05).*

**Table 3:** Metabolomic analysis, relative abundance of phenolic acids, fatty acids/alcohols, phytosterols, and unknown metabolites.

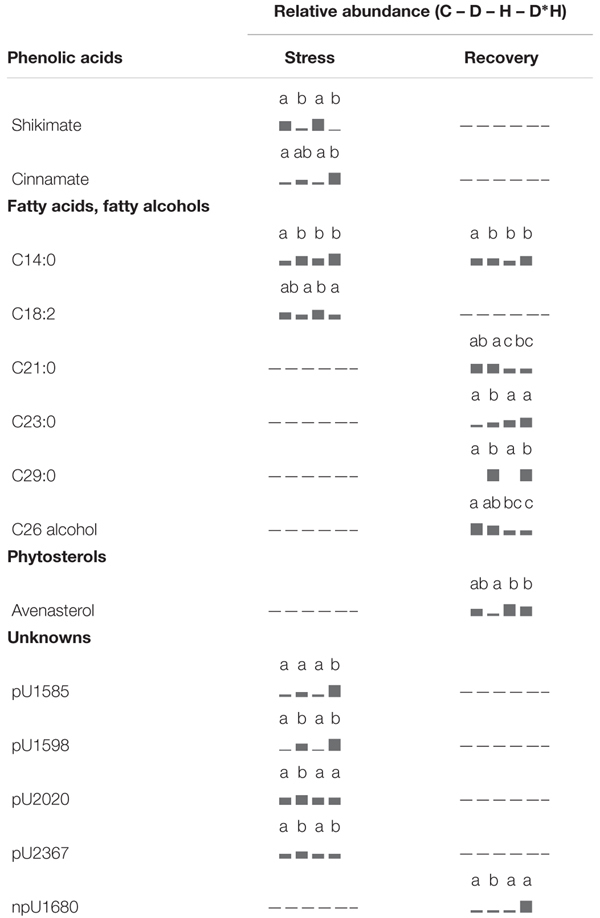

*Abundance data is presented on a scale relative to the lowest value among treatments. Different lowercase letters indicate significant differences between the control (C), drought (D), heat (H), and combined stress (D^∗^H) plant groups, under stress and after recovery; absent relative abundance indicates that no differences were found (*p* ≤ 0.05).*

Regarding carbohydrates (**Table 1**), mannose, galactose and two separate peaks assigned to glucose increased exclusively after drought. Mannitol, sorbitol and inositol contents increased under all stress conditions, and maltose increased in drought and combined stress. On the contrary, abundances of fructose-6-phosphate and glucose-6-phosphate were negatively affected by drought, heat, and combined stress. Dihydroxydihydrofuranone also exhibited decreases under all stress conditions, but with lower magnitude. After recovery, both fructose-6-phosphate and glucose-6-phosphate of heat and combined stressed plants reversed to control levels, but drought stressed plants still kept significantly lower content (**Table 1**). All other carbohydrates reversed the alterations caused by stress after recovery, except inositol that maintained higher levels in plants previously exposed to drought and combined stress (**Table 1**).

Five organic acids – succinate, malate, citrate, quinate, and glycerate – were modulated by stress (**Table 2**). Succinate, quinate, and glycerate abundances were reduced under both drought and combined stress. From these, glycerate was also decreased under heat. Citrate was elevated in response to drought, heat, and combined stress (**Table 2**). Malate abundance was enhanced by drought and reduced after heat, staying unchanged in the combined stress. Following recovery, none of the organic acids showed significant changes relative to control plants (**Table 2**).

Amino acids constitute the largest group of compounds showing significant differences under stress, mainly combined stress (**Table 2**). Aspartate, glutamate, leucine, isoleucine, and proline abundances were significantly increased in drought and heat, and to a greater extent, in the combined stress. Threonine, lysine, histidine, tryptophan, methionine, and GABA were only positively regulated under drought and combined stress, and valine showed an over accumulation only in the combined stress. After recovery, only oxoproline (generated from glutamine during the derivatization procedure), tryptophan and methionine revealed significant alterations (**Table 2**). Oxoproline decreased in previously droughted and combined stressed plants, tryptophan could only be detected in previously drought stressed plants, and methionine decreased in the combined stress, although showing a slight decrease in previously heat stressed plants. Drought, heat, and combined stress positively induced urea levels, which were restored after recovery (**Table 2**).

Regarding phenolic acids (**Table 3**) shikimate decreased under drought and combined stress. Conversely, cinnamate abundance increased under combined stress, although a slight increase was also observed in drought. Neither of these phenolic acids showed significant differences after recovery. Alterations in fatty acids and fatty alcohols were mainly detected in recovery (**Table 3**), with the exception of C14:0, which increased under every stress, and C18:2, which slightly reduced under drought and combined stress. After recovery, C14:0 abundance in previously stressed plants largely reversed to control levels and only plants previously subject to combined stress still presented a significantly higher content. Recovery from drought induced C23:0 and C29:0 accumulation; recovery from heat reduced C21:0 and C26 alcohol; and recovery from combined stress resulted in enhanced C29:0 and reduced C26:0 alcohol. Avenasterol was the only identified phytosterol showing significant changes (**Table 3**); however, the difference observed is due to a lower level of avenasterol on drought in relation to heat and combined stress, representing only a slight decrease when compared with control.

From the unidentified metabolites (**Table 3**), pU2020 increased under drought and pU1585 raised under combined stress, pU1598 and pU2367 increased under both conditions. Moreover, npU1680 revealed an increase after recovery from combined stress.

**FIGURE 8:**
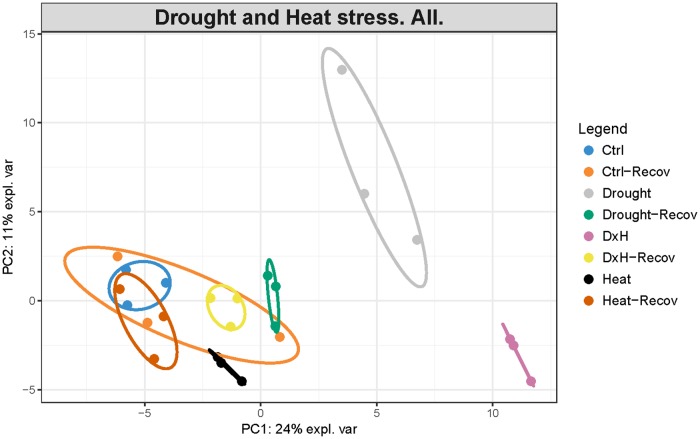
Principal Component Analysis (PCA) of a complete dataset of physiological, biochemical and metabolomic alterations occurring in *Eucalyptus globulus* after several stress conditions (drought, heat, and combined) and recovery. First two components are plotted in the graph. The proportion of variance explained by each component is indicated on axis labels.

The supervised and unsupervised integrated analysis provided us a comprehensive overview of the plant stress responses, identifying the most relevant interactions. Initial comparison based on principal component analysis (PCA) exhibited a clear separation between control and differentially stressed samples in *E. globulus* (**Figure 8**). Control sample scores were grouped together at the bottom left quadrant. Sample scores of plants subject to heat were located more to the right and downwards compared to control, whereas drought sample scores were also placed on the right quadrant but upwards. The combined sample scores were all found together on the bottom right quadrant, farther from the control scores compared to the other stress conditions. The separation between control and heat was mainly related to increased levels of phenylalanine, RWC and some non-polar metabolites, whereas drought separation is mainly driven by accumulation of several sugars (fructose, mannose, glucose), starch, ABA, MDA, and glutathione (Supplementary Table S2). On the other hand, the most separated condition, combined, is mainly driven by increased phenylalanine, RWC and some non-polar metabolites, as seen in heat samples, accumulation of starch, ABA, MDA, and glutathione, as seen in drought samples, together with higher levels of several amino acids (leucine, isoleucine, histidine, tryptophan, asparagine; Supplementary Table S2). After recovery, these differences were mostly reversed and the sample scores of the stressed conditions were found near control sample scores (**Figure 8**).

**FIGURE 9:**
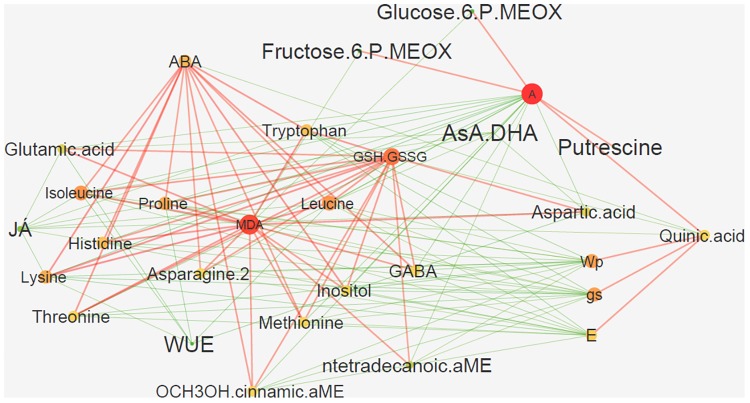
sPLS-based interaction network of the major alterations occurring in *Eucalyptus globulus* after several stress conditions (drought, heat, and combined) and recovery. Network is presented using the force-directed layout, which is based on the “force-directed” paradigm. Color and size of nodes illustrate radiality on a small to large and green to red scale (radiality of a node is calculated by computing the shortest path between the node and all other nodes in the graph); color and size of edges reflect weight with red and green edges illustrating positive and negative correlations, respectively.

The constructed network based on sparse partial least squares (sPLS) allowed the determination of the specific components behind the observed phenotypical changes considering the complete metabolomic, biochemical and physiological changes of the experiment (**Figure 9**). This network highlighted photosynthesis (A), MDA, glutathione (GSH.GSSG) and ascorbate (AsA.DHA) as biochemical central points, which are positively and negatively correlated with the studied hormones (ABA and JA) through several metabolomic alterations (**Figure 9**). Specifically, JA and AsA.DHA are negatively correlated with glutamic acid, isoleucine, lysine, aspartic acid, proline, and tryptophan (**Figure 9**). Most of these are positively correlated with glutathione and MDA and also mediating a positive correlation between these and other amino acids (GABA, histidine, leucine), and ABA. Finally, another key interaction places A with a positive relation with putrescine, glucose-6-phosphate, fructose-6-phosphate, and quinic acid (**Figure 9**). This last one, quinic acid, also mediates a positive interaction between A, on one side, and Ψ_pd,_ E and g_s_, on the other. All of these reveal negative interactions with most of the intervening amino acids.

## Discussion

Considerable research advances have been accomplished focusing on plant responses to single stress factors under controlled environments (Mittler and Blumwald, 2010). However, plants growing in the field encounter a number of different co-occurring abiotic stresses that most probably cannot be extrapolated by the sum of the different stresses applied individually, altering plant metabolism in a novel manner (Rizhsky et al., 2002; Zandalinas et al., 2016). Bearing this in mind, we aimed to determine convergent and divergent responses of the individual stresses in relation to their combination, evaluating the impact of drought and heat stress (alone and in combination) and respective recovery using a drought-tolerant *E. globulus* clone.

Regarding drought stress alone, the main responses included reduced Ψ_pd_, gas exchange, JA, fructose-6-phosphate, glucose-6-phosphate, α-glycerophosphate, and shikimate, and increases in MDA, glutathione pool, ABA, amino acids, starch, and non-structural carbohydrates. Most of these results are in agreement with other reports that analyzed the isolated effect of drought on *E. globulus* (Warren et al., 2011; Correia et al., 2014b, 2016a,b), and indicate that water deficit negatively affects plant water relations and photosynthesis, causing a moderate oxidative stress, and inducing enhanced osmoprotection and other defence-related pathways.

On the other hand, heat stress alone triggered an increase in RWC, Ψ_pd_, mannitol, sorbitol, inositol and several amino acids that were accompanied by a reduction in the photosynthetic rate and pigments, WUE, starch, fructose-6-phosphate, glucose-6-phosphate, α-glycerophosphate, SA, and JA. The reduction in the photosynthetic rate and pigments in parallel with unaffected transpiration rate and stomatal conductance confirms the particular sensitivity of photosynthesis to heat stress (Sharkey, 2005), even in a short heat shock (4 h at 40°C). It also indicates that the main limitations are non-stomatal and mostly related to heat-induced alterations in enzyme activity (Larkindale et al., 2005). A decrease in photosynthetic pigments, fructose-6-phosphate, glucose-6-phosphate, and starch was also documented in potato leaves growing at a moderately elevated 30°C (Hancock et al., 2014). No major oxidative impairment was detected and this can be explained by the shifts in the polyols mannitol, sorbitol, inositol and several amino acids, such as proline, possibly indicating that these compatible solutes were effective hydroxyl radical scavengers (Smirnoff and Cumbes, 1989; Wang et al., 2003). In addition to their role as radical scavengers, the accumulation of the polyols under heat stress is most likely responsible for the observed increase in RWC and Ψ_pd_, reinforcing their primary role as osmoprotectants (Bokszczanin et al., 2013).

The heat-induced reduction in SA and JA is an unexpected result since both hormones are reported to play an important role as signal molecules in abiotic stress tolerance (Horváth et al., 2007; Xu et al., 2016) and SA has been reported to protect plants from heat stress (Wang et al., 2014). However, the downregulation of JA has already been described in *E. globulus* under water deficit (Correia et al., 2014a). Our results further confirm the downregulation of JA under drought stress, highlighting a similar response triggered by heat stress regarding not only JA but also SA. The way these abiotic stresses influence these two phytohormones in *E. globulus* is yet to be discovered. However, SA and JA, together with ethylene, are known to play major roles in regulating plant defense. SA is usually associated with the activation of defense against biotrophic and hemi-biotrophic pathogens, and the establishment of systemic acquired resistance (SAR). JA and ethylene are generally involved in defense against necrotrophic pathogens and herbivorous insects [reviewed by Bari and Jones (2009)]. Hence, this result has significance in terms of the impact of abiotic stress on biotic interactions, suggesting that these abiotic stresses can negatively influence defense against other biotic threats.

A divergent response between isolated drought and heat stress is related to changes in the TCA cycle intermediates. In heat, citrate increase went along with reduced malate, whereas drought-induced increases of citrate and malate were accompanied by reduced succinate. Together with the different amino acids that accumulate in each stress this result highlights two different metabolic regulations. In heat, the TCA cycle flux appears to be changed to two weakly connected branches, with malate functioning as a mitochondrial respiratory substrate to produce citrate, which is then converted to glutamate and proline. Similar cases of the non-cyclic flux mode of TCA cycle has been reviewed elsewhere (Sweetlove et al., 2010). However, the prevailing pathway activated under this condition appears to be the shikimic acid pathway, revealed by the over accumulation of shikimate and phenylalanine. Conversely, the shikimic acid pathway is downregulated under drought conditions. In this stress scenario, an induction in the first steps of the TCA cycle likely supplies higher demands for citrate that is metabolized to amino acids of the glutamate family; and succinate is converted to malate, which in turn is redirected to produce amino acids of the oxaloacetate/aspartate family.

Still on this subject, comparing the isolated stresses with the combined one reveals novel responses. In the combined conditions of drought and heat stress, the highest accumulation of citrate was accompanied by reduced succinate without major alterations in malate. The higher content of α-glycerophosphate together with the major accumulation in amino acids of the glutamate family, the oxaloacetate/aspartate family and leucine/valine indicates that glycolysis is enhanced in this combined condition, sustaining the higher demand for amino acids. Still, we should also note the possibility for amino acid mobilization resulting from protein breakdown as protein turnover has been described as an important regulatory mechanism that allows plant cells to respond to drought and recovery (Lyon et al., 2016). The absence of significant changes in the fatty acids/alcohols and phytosterols detected at this new stress state does not support the premise of a regulation by changes of membrane lipids as we could assume (Falcone et al., 2004).

A novel response triggered only by the combined effect of drought and heat was the induction of cinnamate. Cinnamate originates all phenylpropanoids through the action of phenylalanine ammonia-lyase (PAL) on phenylalanine (Dixon and Paiva, 1995). We are yet uncertain of which phenylpropanoids are generated under this condition since a number of different phenylpropanoids can be involved (Dixon and Paiva, 1995).

Drought-stressed plants subject to a heat shock revealed a decrease in gas exchange (sharp), WUE, Ψ_pd_ and JA, no alterations in EL, MDA, starch and pigments and increased glutathione pool in relation to control. Comparing with drought stress alone, this reveals that subjecting drought stressed plants to an additional heat stress alleviated Ψ_pd_ and MDA, maintaining an increased glutathione pool and reducing starch content and non-structural carbohydrates. Interestingly, and in contrast to the expected negative effect of the stress combination on plant growth reported for other species (Silva et al., 2010), these results highlight that the combination of drought and heat provides significant protection from more detrimental effects of drought-stressed eucalypts. A similar conclusion has been described for tomato plants under the combined effect of salinity and heat (Rivero et al., 2014).

Regarding recovery, most of the parameters affected by each stress condition reversed after re-establishment of the control growing condition. This is a common reported response (Correia et al., 2014a,b, 2016b; Escandón et al., 2016). Gas exchange and some carbohydrates reversed at a slower pace after drought and combined stress, which reveals the sensitivity of the photosynthetic apparatus (Chaves et al., 2009) and points out the most restrictive effect of these two stress conditions. On the other hand, the different modulation of several fatty acids/alcohols and phytosterols after recovery from drought and combined stress uncovers a putative regulation that allows restoration after stress through changes of membrane lipids (Falcone et al., 2004).

In accordance with the idea that relatively few studies have attempted to correlate metabolite content with physiological data, and the advantages of those (Tohge et al., 2015), we decided to introduce an integrative approach to analyze our dataset. The PCA and network results summarize the overall knowledge acquired in our study, aligning with some regulatory networks already described for their involvement in tolerance and recovery to drought (Brossa et al., 2015; Lyon et al., 2016), as well as other stresses (Das and Roychoudhury, 2014; Xu et al., 2015).

At present, information on the combined effect of heat and drought stress in *Eucalyptus* is rather limited although much needed from the application point of view (e.g., finding suitable markers for selecting the most tolerant genotypes to field establishment). In this work, we have reported different physiological, biochemical and metabolic adjustments that enable *E. globulus* to thrive under conditions of drought and heat applied alone or in combination. Although a few mechanisms were convergent to all stress conditions, the response magnitude was very dependent on the specific stress, and most of the metabolic pathways responded uniquely to each specific stress. Rather than presenting an additive outcome, the combination of heat stress ameliorated part of the negative effect of drought. The information collected here confirms that the biological processes switched on by an environmental factor are very specific to that exact condition and are likely to differ from those activated by a slightly different environmental condition (Mittler, 2006; Atkinson and Urwin, 2012). The need for studies that focus on the actual field stress conditions is thus evident and imperative for selecting plants with enhanced tolerance to naturally occurring environmental conditions.

## Author Contributions

GP designed and supervised the experimental procedure. RDH designed and supervised the biochemical and metabolomic characterization. AG-C designed and supervised the hormonal quantification. BC, JA, and GP performed the experiment, the physiological, and the biochemical characterization. BC and RDH performed the metabolomic profiling and analysis. LV designed and performed the PCA and network analysis. BC, RDH, and GP wrote the manuscript. All authors discussed the data and reviewed the manuscript.

## Conflict of Interest Statement

The authors declare that the research was conducted in the absence of any commercial or financial relationships that could be construed as a potential conflict of interest.
